# Correlative atomic force microscopy quantitative imaging-laser scanning confocal microscopy quantifies the impact of stressors on live cells in real-time

**DOI:** 10.1038/s41598-018-26433-1

**Published:** 2018-05-29

**Authors:** Supriya V. Bhat, Taranum Sultana, André Körnig, Seamus McGrath, Zinnat Shahina, Tanya E. S. Dahms

**Affiliations:** 1 Department of Chemistry and Biochemistry, University of Regina, 3737 Wascana Parkway, Regina, SK S4S 0A2 Canada; 2 JPK Instruments, JPK Instruments AG, Colditzstr. 34-36, 12099 Berlin, Germany

## Abstract

There is an urgent need to assess the effect of anthropogenic chemicals on model cells prior to their release, helping to predict their potential impact on the environment and human health. Laser scanning confocal microscopy (LSCM) and atomic force microscopy (AFM) have each provided an abundance of information on cell physiology. In addition to determining surface architecture, AFM in quantitative imaging (QI) mode probes surface biochemistry and cellular mechanics using minimal applied force, while LSCM offers a window into the cell for imaging fluorescently tagged macromolecules. Correlative AFM-LSCM produces complimentary information on different cellular characteristics for a comprehensive picture of cellular behaviour. We present a correlative AFM-QI-LSCM assay for the simultaneous real-time imaging of living cells *in situ*, producing multiplexed data on cell morphology and mechanics, surface adhesion and ultrastructure, and real-time localization of multiple fluorescently tagged macromolecules. To demonstrate the broad applicability of this method for disparate cell types, we show altered surface properties, internal molecular arrangement and oxidative stress in model bacterial, fungal and human cells exposed to 2,4-dichlorophenoxyacetic acid. AFM-QI-LSCM is broadly applicable to a variety of cell types and can be used to assess the impact of any multitude of contaminants, alone or in combination.

## Introduction

There has been an exponential increase in the release of anthropogenic pollutants, such as pesticides^1^, toxins^2^, pharmaceutical drugs^3^, personal care products^4^, microbeads^5^, and nanoparticles^6^, into our environment. The negative consequences of these contaminants are often discovered in hind sight since we currently lack appropriate tools to assess their effects at the cellular level^7^ prior to their release. It is therefore imperative to assess the impact of these chemicals, alone and in combination, on human health^8^ and the microbial environment^9^. New technologies to directly visualize cellular behaviour and processes (“cellulomics”) are continually being developed, ideally with the ability to study dynamic processes in live cells with minimal damage. The utility of any technique is augmented when combined with other methods. All microscopes, from optical to electron and surface scanning, have associated limitations that can be partially overcome by correlative microscopy: the correlation of data collected separately on individual microscopes or that from fully integrated microscopes during simultaneous imaging^10^. The physical integration of two or more microscopes into a single more powerful instrument to overcome individual limitations is becoming the new norm, ideally producing highly resolved images of biological specimens containing both structural and compositional information^11^.

Simultaneous multi-mode imaging using an atomic force microscope fully integrated with a confocal laser scanning microscope has interpretive value that is greater than the sum of its parts, offering wide spatial resolution ranges (nm-mm), high temporal (ms) resolution paired with sensitivity to local chemistry and functional analysis^12^. Atomic force microscopy (AFM) images the surface ultrastructure and probes mechanical properties with nm- and pN scale resolution, respectively, and this is complimented by the optical sectioning capabilities, excellent temporal resolution and high contrast of confocal microscopy (CM) ^12^. AFM not only reports on cell surface ultrastructure and remodelling (topography), but can be used to map (adhesion) surface molecules^13^, or probe cellular integrity (viscoelasticity), all of which relate to overall cellular health, for example cancer progression^14,15^ and metastasis^16^. Since conventional AFM and CM are temporally^17^ and diffraction^18^ limited, respectively, and both are capable of studying live cells under physiologically relevant conditions, they make an excellent pair for physical integration and routine simultaneous correlative imaging. Integrated AFM-LSCM reports on spatio-temporal events with high sensitivity, from the cell surface to its depth, to provide unprecedented insight into intricate cellular events^12^.

**Figure 1:**
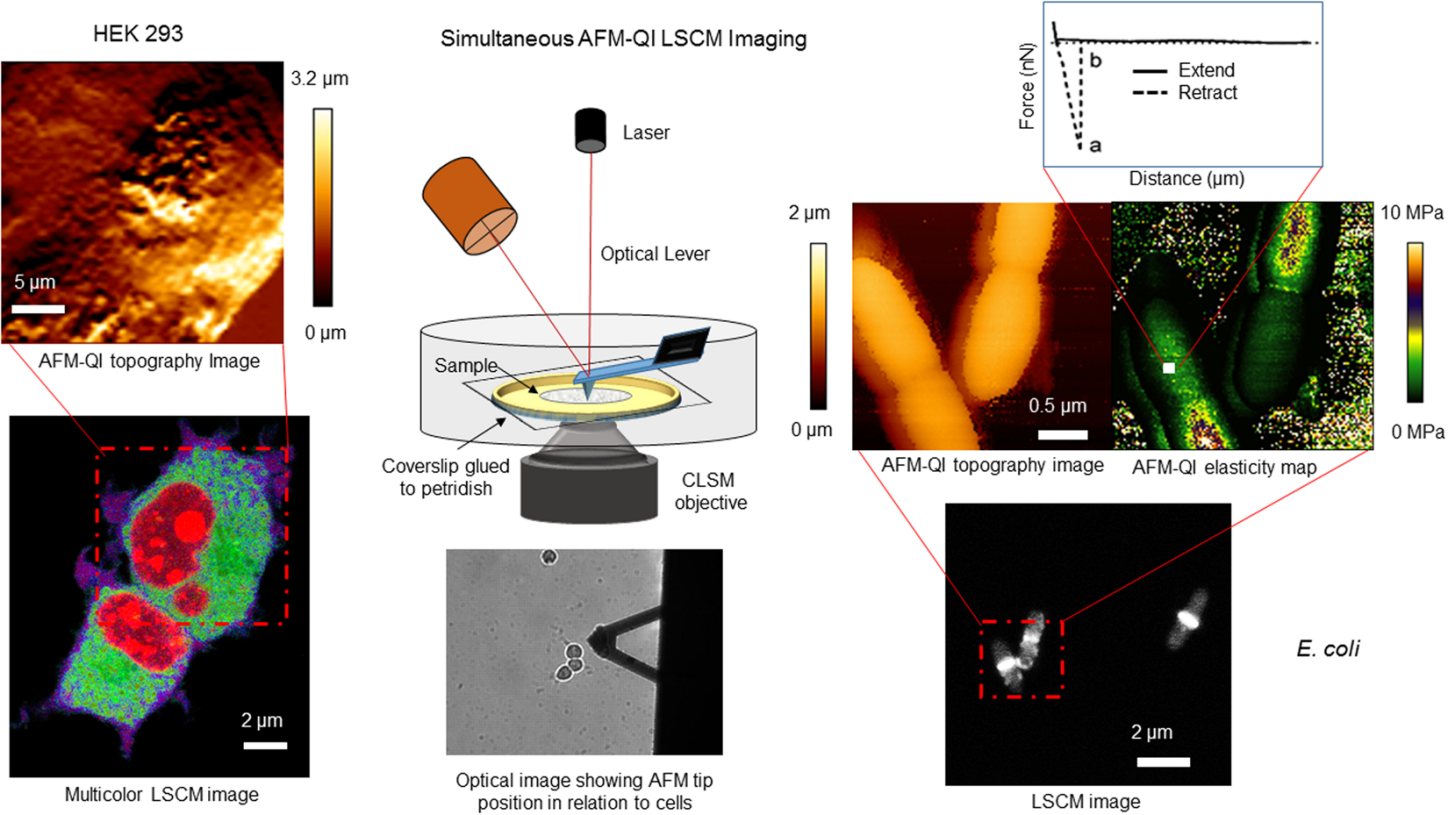
AFM-QI-LSCM schematic illustration showing simultaneous data collection of multiplexed data. A live biological sample firmly immobilised to a substrate is mounted on a petridish. The inverted LSCM objective is focused onto the sample from below with the AFM cantilever and tip imaging from above. AFM-QI provides information on sample surface topography, (visco) elasticity and adhesion while LSCM provides localization of multiple fluorescently tagged molecules within the sample. Simultaneous AFM-QI-LSCM imaging provides high content data on live, actively dividing biological samples in an enriched aqueous environment.

AFM has been correlated with LSCM, including total internal reflection^19^, to identify viral binding events^19–21^ on live cells, mechanical stimulation^22^ and nano manipulation^23–25^ of live mammalian cells. The advent of super resolution optical imaging has also made correlation with AFM possible in live human cells^26,27^ with the potential for single molecule detection from each^28,29^. AFM integrated with inverted epifluorescence microscopy has been used to observe *Candida*-macrophage interactions^30^, and AFM-LSCM to view changes in morphology and viability for solvent exposed bacteria^31^. However, correlative AFM-LSCM has never successfully produced routine, high content data for live, actively growing cells, in particular bacteria^32^, to assess their response to environmental contaminants in real-time (Fig. 1). The quantitative imaging (QI^TM^) mode of AFM collects force-distance curves at every pixel in a high resolution image, unlike the traditional method requiring two steps for high resolution AFM topography and then low resolution ‘force mapping’. This multiparametric mode produces force curves with information on height, surface stiffness and adhesion at each high resolution pixel. The absence of lateral forces facilitates imaging of biological and loosely attached samples, for example bacteria, as it reduces the probability of sample detachment^33^.

Herein we demonstrate for the first time simultaneous AFM-QI-LSCM imaging of living bacteria, yeast and mammalian cells responding to the herbicide 2,4-dichlorophenoxyacetic acid (2,4-D) *in situ*, with the continuous monitoring of morphology, surface ultrastructure, mechanical properties, adhesion and tracking multiple fluorescently tagged molecules in real time. The novel use of this method to probe *Escherichia coli*, *Candida albicans* and HEK 293 cells in response to a xenobiotic during active cell division highlights the versatility of the method, with future broad application for assessing the impact of virtually any type of anthropogenic contaminant on most cell types.

## Results

**Figure 2:**
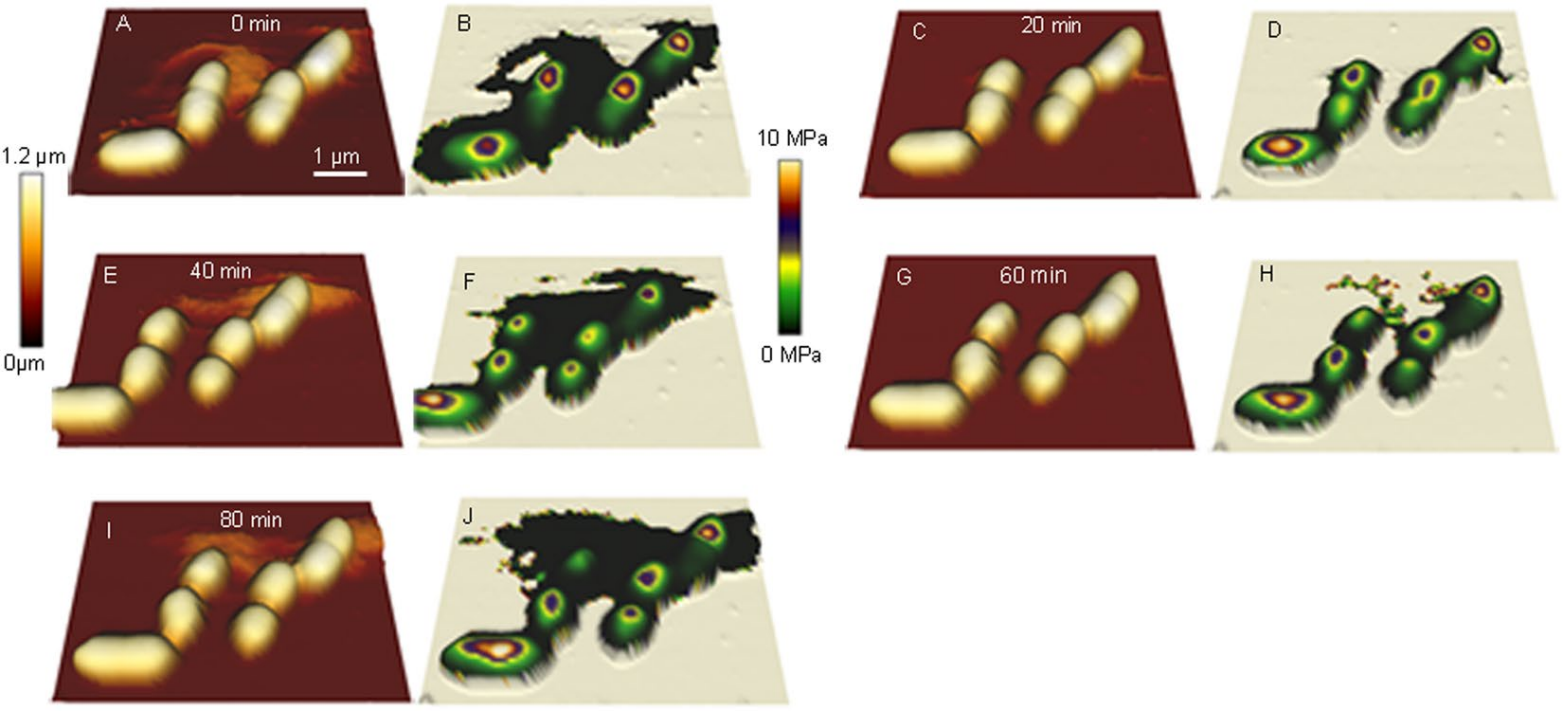
AFM-QI time lapse images showing topography and Young’s moduli during cell division. Height images (**A**,**C**,**E**,**G** and **I**) clearly show various stages of septum formation and separation of daughter cells, whereas QI maps (**B**,**D**,**F**,**H** and **J**) probe changes to surface elasticity. Elasticity was unaltered during cell division, and only elasticity values from the middle of cells were considered accurate due to artifacts at cell edges.

It was important to characterise changes to cell morphology, surface ultrastructure and physical properties under conditions favorable for *E*. *coli* proliferation to produce data relevant for assessing changes associated with exposure to stress. Actively dividing *E*. *coli* WM1074 were imaged by AFM-QI to produce time lapse images, showing every step of cell division, including cell elongation, initiation of constriction at the mid cell, extension of constriction and separation of daughter cells at high resolution (Fig. S1 and Movie S1). Following division, some cells detached, became planktonic and swam/floated away in the middle of imaging. It is to be expected that the Cell-Tak used to immobilize the parent cell during sample preparation is no-longer effective after multiple cell divisions, allowing for cells to become planktonic. At every step of the cell division process, Young’s moduli and adhesion could be extracted from the *E*. *coli* QI images, showing a spectrum of changes in elasticity (Fig. 2) across their surface. The center of the cell had a much higher elasticity (1–1.5 MPa) when compared to the “apparent elasticity” at edges (200–300 KPa) for all samples, regardless of the imaging media. The apparent elasticity is an artifact caused by the side of the tip contacting the steep cell edge, and possibly also a slight displacement of the cell as a result of imaging. *E*. *coli* surface adhesion, a result of tip-sample interactions, did not vary over cell division but varied slightly in different imaging media, with the highest adhesion to the silicon nitride tip observed in 0.01 M PBS and the least in pure LB (Table 1). There was no significant change in elasticity and roughness with media type, suggesting that dilution of the media had no significant impact on surface molecular organization. Cell division was slower (~2.5 h) in PBS, but approximately every 20 min in dilute and full strength LB, so diluting the media in half with PBS did not impact doubling time. Some cells detached and floated away after several divisions (Movie S1), but those that remained immobilized formed microcolonies through continuous division (Fig. S2), for which the surface properties probed by AFM remained the same.

**Table 1:** Young’s moduli, adhesion and roughness for *E*. *coli* in different media and for *E*. *coli*, *C*. *albicans* and HEK 293 exposed to 2,4-D.

Organism, Strain, Conditions	Young’s modulus (MPa)	Adhesion (pN)	Roughness (nm)
*E*. *coli* WM1074			
PBS	1.21 ± 0.06	380 ± 20*	15.9 ± 5.8
LB	1.06 ± 0.35	160 ± 7*	16.8 ± 6.9
PBS/LB	1.5 ± 0.62	280 ± 10*	17.2 ± 6.0
PBS/LB + 2,4-D^33^	0.29 ± 0.16*	360.0 ± 29.6*	22.1 ± 12.2*
*C*. *albicans* RSY150			
YPD	0.13 ± 0.05	108 ± 20	61.3 ± 0.3
YPD + 2,4-D	0.28 ± 0.11*	200 ± 90*	38.0 ± 6.7*
HEK 293			
DMEM/FBS	0.0005 ± 0.0002	210 ± 50	346.2 ± 48.7
DMEM/FBS + 2,4-D	0.0003 ± 0.0001*	370 ± 30*	296.8 ± 49.7*

Changes that are significant (p < 0.05) are indicated by an asterisk.

**Figure 3:**
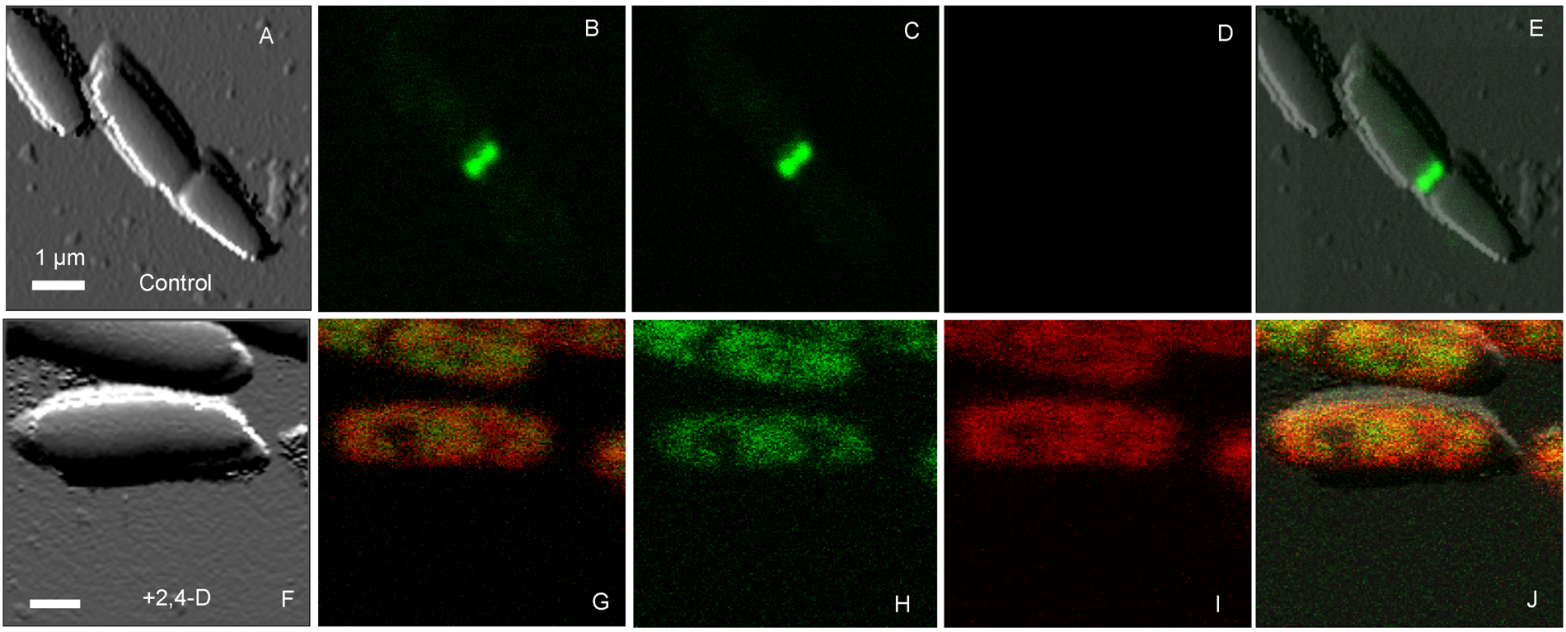
Simultaneous AFM-QI-LSCM of *E*. *coli* showing the localization of the Z-ring and onset of ROS during 2.4-D exposure. (**A**–**E**) Show the presence of a distinct Z-ring (**C**) in control cells and absence of a ROS signal (**D**). (**F**–**J**) Show the immediate delocalization of the Z-ring resulting in a diffuse green fluorescence and an increase in ROS signal. The confocal image was collected 5 min following the addition of 1 mM 2,4-D directly into the imaging media. (**E** and **J**) Are AFM images overlaid with the confocal images. FtsZ-GFP is shown in green and ROS, labeled with Cell-ROX, is shown in red. G and B show localization of two colors simultaneously. Bar is 1 µm.

WM2026 with FtsZ-GFP imaged by LSCM further confirmed active cell division, as denoted by a distinct dynamic Z-ring at mid-cell (Fig. 3A–E). Cell-ROX dye added to the WM2026 media, approximately 30 min before the addition of 1 mM 2,4-D, gave a ROS signal (Fig. S3) that was accompanied by loss of the Z-ring (Fig. 3F–J). AFM-QI-LSCM was used to simultaneously track the ROS and FtsZ-GFP signals, along with changes to surface adhesion and elasticity following the addition of 2,4-D. Further detail on the impact of 2,4-D in *E*. *coli* is described in our previous work^34^.

**Figure 4:**
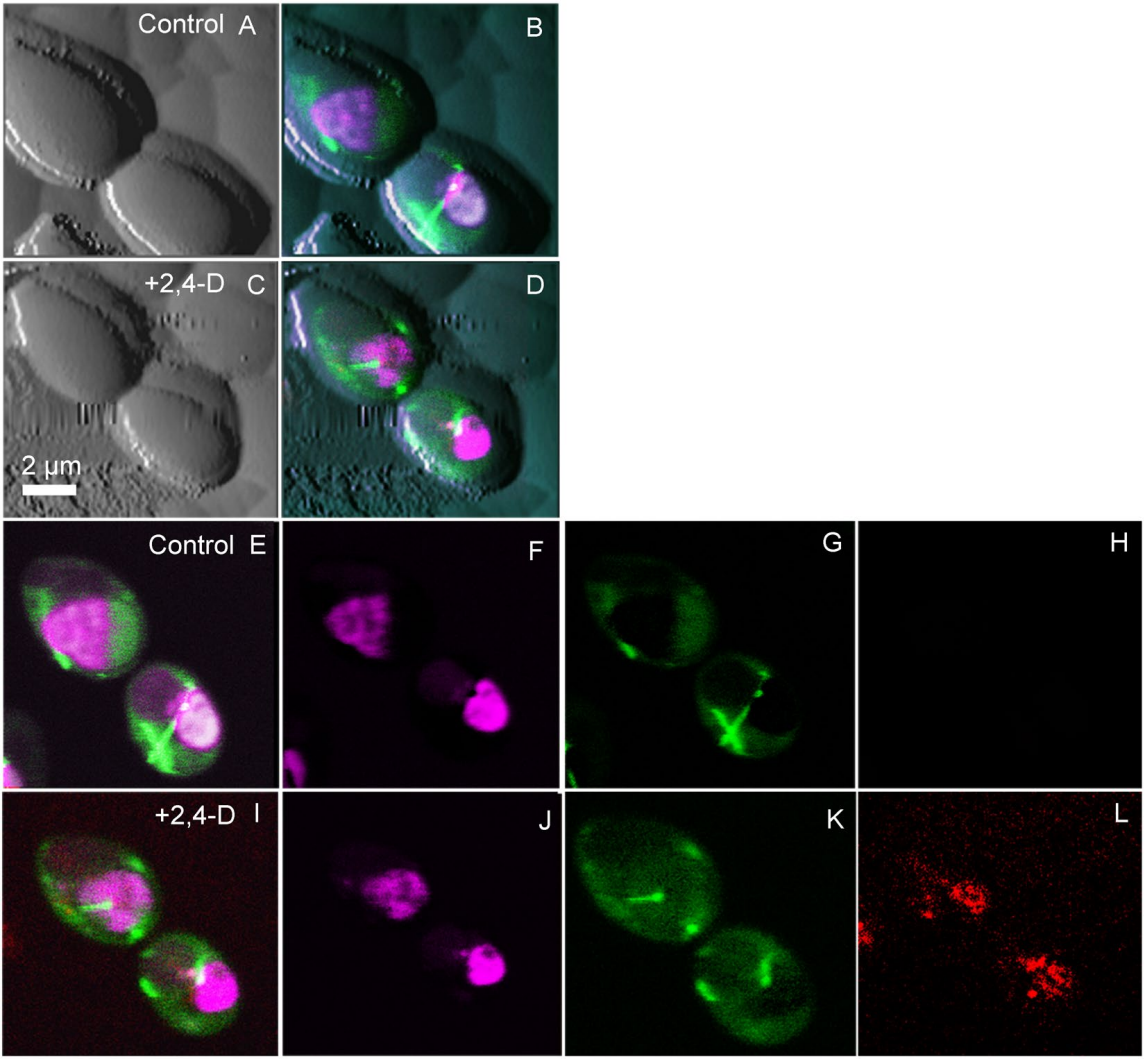
Simultaneous tricolor AFM-LSCM images of *C*. *albicans* showing the localization of tubulin2-GFP (Tub2-GFP, green), histone protein B-RFP (Htb-RFP, purple) and onset of ROS (Cell-ROX-deep red, red) after 2,4-D exposure. (**A**–**D**) Show multicolored LSCM images overlaid onto the AFM QI image before (**A**,**B**) and after (**C**,**D**) 2,4-D exposure. Confocal images were taken at mid-cell, whereas AFM quantitative images were at the surface, making the two images look very different. (**E**–**H**) Show control images taken before the addition of 8 mM 2,4-D, indicating the absence of a ROS signal (**H**). (**I**–**L**) Are images collected 5 min after the addition of 2,4-D, clearly showing a ROS signal (**L**). (**E** and **I**) Show tricolour merged images with Tub2-GFP, Htb-RFP and Cell-ROX, F and J show only RFP, G and K show only GFP and H and L show only ROS.

Using a similar strategy, AFM-QI-LSCM of *C*. *albicans* RSY150 co-expressing tubulin2-GFP (Tub2-GFP) and histone protein B-RFP (Htb-RFP) showed an increase in ROS within minutes (Fig. S3), but at much higher concentrations of 2,4-D, along with a significant reduction in surface roughness, and a two-fold increase in both surface adhesion or elasticity (Table 1). Even at 8 mM 2,4-D exposure, there was no significant change in tubulin2 or histone distribution (Fig. 4).

**Figure 5:**
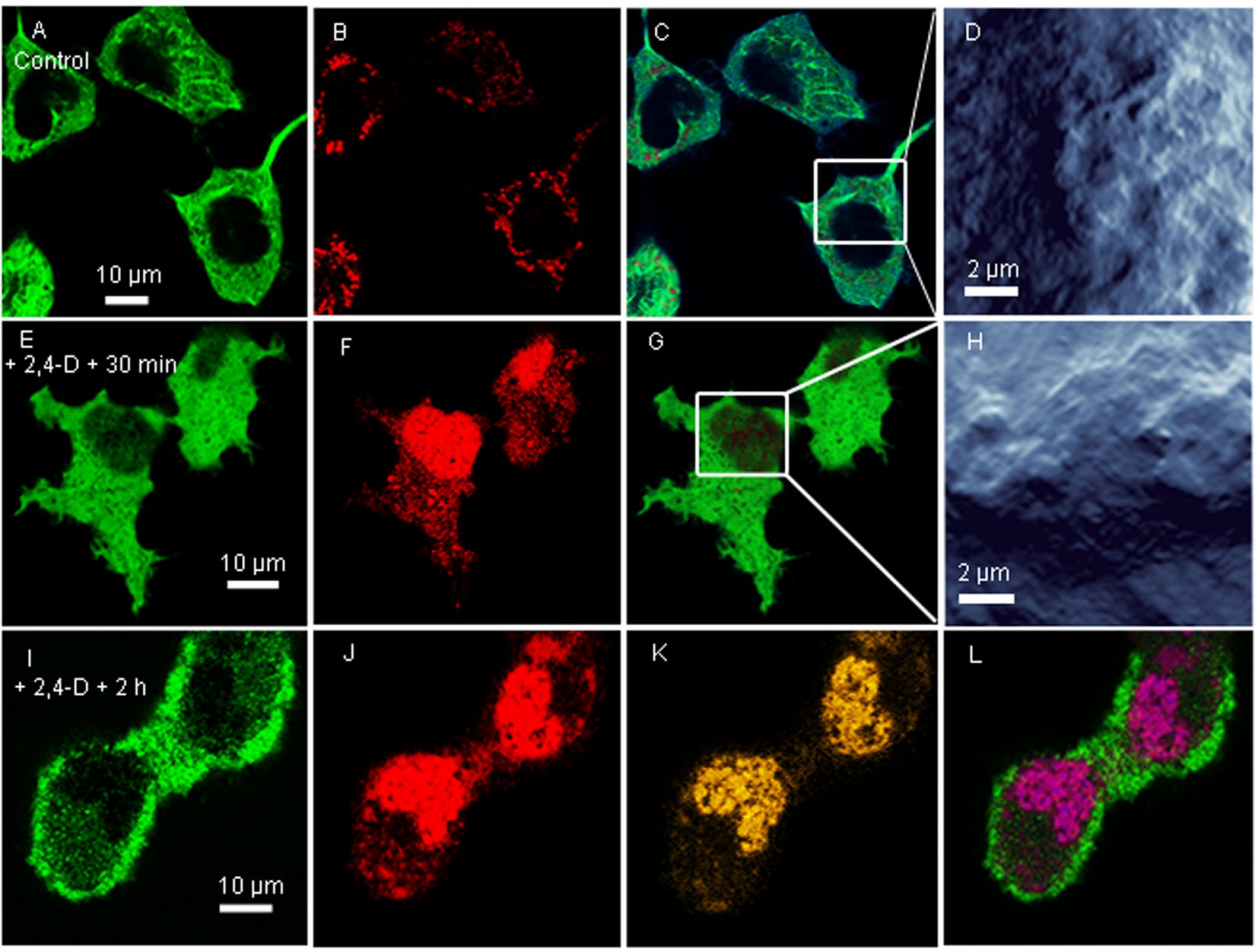
Simultaneous tricolor AFM-LSCM images of HEK-293 with GFP-tubulin (green), Cell-ROX (red) and Syto 82 nuclear dye (orange) showing morphological changes and altered localization of ROS after 2,4-D exposure. In the absence of 2,4-D (**A**–**D**), the tubulin network is clearly visible with a low ROS signal (red) localized to the mitochondria. The ROS signal increased in intensity after the addition of 1 mM 2,4-D (**E**–**H**) and was eventually mainly localized to the nucleus. The surface ultrastructure (D versus H) and properties (Table 1) were altered with 2,4-D exposure. Following 2 h exposure, the cells lost their protrusions, appeared rounded and more closely associated with neighbouring cells (**I**–**L**). (**C** and **G**) Are dual color images showing tubulin and ROS, and L is the triple color image showing all three signals, with convolution of orange and red signals shown in purple.

HEK 293 cells, over 7 µm tall, sometimes displayed noise as a result of interference between the top of the cells and cantilever holding the AFM tip. In the absence of 2,4-D, HEK 293 cells imaged by AFM-QI-LSCM had a smooth surface, a clear tubulin-GFP network, a distinct nucleus, and a very low intensity ROS signal (Fig. 5A–D) localized to the mitochondria. The addition of 1 mM 2,4-D directly into the imaging medium caused a slow loss of the GFP signal over 30 min, an increase in mitochondrial ROS signal within 30 min (Fig. S3) and no change in Syto 82 intensity or localization (Fig. 5L). After the addition of 2,4-D, the ROS signal, initially localized to the mitochondria, intensified in the nucleus within minutes (Fig. 5E–H). Exposure of HEK 293 to 2,4-D caused a complete disruption of the tubulin network, which delocalized throughout the cytosol, accompanied by a significant increase in adhesion, decrease in Young’s Modulus and roughness after 30 min. Cells shrank, became rounded, lost their normal extracellular protrusions (Fig. 5I–L) and detached from the surface after 2 h.

## Discussion

We have demonstrated for the first time correlative AFM-QI-LSCM for the acquisition of nm-resolution surface ultrastructure, pN-scale nanomechanics and simultaneous localization of two and three intracellular signals in actively dividing bacterial, fungal and human cells in real-time (Fig. 1). Cells assessed for their response to 2,4-D exposure included a prokaryote (*E*. *coli*) and two different eukaryotes (the yeast *C*. *albicans* and human HEK 293 cells), showing the broad applicability of the method for assessing the impact of stressors in a wide range of cell types.

AFM QI mode is advantageous when imaging challenging biological samples that are soft and sticky, providing nano-mechanical and topographical maps of the entire surface^33^. QI enabled the imaging of loosely bound samples, for example actively diving bacteria that were detaching and becoming planktonic. We collected complete surface maps at various stages for actively dividing live *E*. *coli* (time lapse Fig. 2) over a period of several hours. Artifacts in height measured at edges are caused by collision between the sample and cantilever holding the AFM tip, and can be addressed by using thinner, very long (~7 µm) tips, even during high speed imaging^17^. The latter phenomenon combined with the Hertz fit, which assumes indentation of a completely elastic sample^35^, gives rise to apparent elasticity values at edges where there is not only indentation, but likely a slight displacement of the sample. There was no change in elasticity and adhesion during various stages of *E*. *coli* cell division, indicating that the cell surface composition at central regions remains relatively constant over the *E*. *coli* life cycle. Changes in tip-sample adhesion with imaging media relates to the differing ionic nature of the solutions. The greatest adhesion to the silicon nitride tip was observed in pure PBS due to a combination of increased electrostatic and steric interactions. Since the surface of the Si_3_N_4_ tip is zwitterionic-like^36^, adhesion to the highly anionic polysaccharides on the *E*. *coli* surface^37^ is enhanced by bridging, which is more prevalent at higher ionic strengths^38^. Adhesion, even when non-specific, is an important comparative characteristic that reflects the macromolecular arrangement of the cell surface which can impact important physiological processes such as biofilm formation and host cell invasion^9,33,34,39–41^.

To demonstrate proof-of-principle and dramatic responses during the correlative imaging assay, we chose *E*. *coli* exposed to the chemical stressor 2,4-D, which we previously showed to induce filamentation, ROS, the SOS response, cell envelope remodelling, along with the relocalization of components of the cell division machinery (FtsA, FtsZ)^34^. As expected, *E*. *coli* exposed to 2,4-D for 30 min had a higher ROS signal (Fig. S3), disruption of the Z-ring, changes to surface ultrastructure, elasticity and adhesion (Fig. 3), consistent with our previous study^34^.

*C*. *albicans* was selected as a test organism since yeast shares with *E*. *coli* an oxidative stress response to 2,4-D^42,43^, but adapts in a very different way, with halted, then slowed cell division, multidrug resistance, an increase in saturated membrane fatty acids^44^ and transcriptome-wide changes^42^. Indeed, we observed an increase in ROS for *C*. *albicans* exposed to 2,4-D, a remodelled cell surface with a two-fold increase in adhesion and elasticity (Table 1), along with a smoother exterior (Fig. 4). The latter is in contrast to the increase in surface roughness of *C*. *albicans* in response to cinnamon bark oil^45^, curcumin^46^, caspofungin^47^, all of which target the cell wall and initiate cell wall integrity pathways, flucytosine which targets DNA and RNA synthesis^48^, or the membrane targeted amphotericin B^48^. The unique cell wall remodelling with 2,4-D exposure likely reflects reorganization of membrane fatty acids and breakdown of the membrane permeability barrier in yeast^44^. We did not expect 2,4-D to alter the histone distribution in yeast, but possibly tubulin2 since 2,4-D disrupts tubulin polymerization in other eukaryotes^49^ and yeast tubulin2 is the structural analog to FtsZ in bacteria^50^. However, there was no change in the tubulin2 signal with 2,4-D, demonstrating the difference in eukaryotic responses and those between yeast and bacteria. Yeast such as *Saccharomyces* and *Candida* possess a thick envelope that likely prevent the entry of 2,4-D, changing its fate and consistent with the higher MIC^51^.

Cytotoxic 2,4-D leads to apoptosis in a variety of human cells, and in particular HEK 293 cells^52–54^. The addition of 2,4-D resulted in the loss of the microtubule network (Fig. 5), with the tubulin signal slowly fading away, consistent with prior studies showing 2,4-D disrupts microtubule stability in human lung cells^50^ and cultured neurons, and inhibits *in vitro* tubulin polymerization^49^. The appearance of smaller, rounder cells lacking extracellular protrusions, which led to detachment, indicates loss of the microtubule network during 2,4-D exposure also consistent with an apparent loss of cell elasticity indicated by the lower Young’s modulus (Table 1). Constitutive low levels of ROS in HEK 293 mitochondria is consistent with the byproducts of oxidative phosphorylation^55^. Post 2,4-D exposure, HEK 293 showed oxidative stress, with the ROS signal initially growing in intensity in the mitochondria and then appearing in the nucleus. This is all consistent with immediate uncoupling of oxidative phosphorylation in the mitochondria^56,57^, followed by an oxidative stress response which leads to a leaky nuclear barrier, and finally DNA damage^58,59^, which occurs more slowly as a result of nuclear mechanisms that protect DNA from oxidative damage^60^. Nonviable HEK 293 cells following the initial mitochondrial ROS burst are consistent with “ROS-induced ROS release” from adjacent mitochondria meant to amplify oxidative stress and promote cell death^61^, which would also explain the delayed nuclear ROS signal.

Simultaneous correlative microscopy demands the full integration of two or more microscopes, in this case atomic force and laser scanning confocal. Commercially available instruments are designed to be compatible, but need to be integrated in house. Pre-calibration and post-processing with the AFM software (JPK) has been developed to ensure that final images are in direct correspondence. Even for this fully integrated instrument with multiple detectors, the different time scales of each microscopic mode presented challenges for simultaneous imaging and required time syncing of images post processing (reviewed in^12^).

Software and user interfaces available for proper simultaneous AFM-LSCM measurements and image overlay are limited, with few improvements since Gradinaru *et al*. developed a home-built integrated atomic force and two-photon fluorescence instrument with single molecule sensitivity in 2004^62^. High speed (HS) AFM^17,63^, lower noise systems with improved instrument integration and improved models to better interpret the results will further enhance the application of this technique. Biochemically modified AFM tips^13^ make it possible to map or stimulate single molecules on the cell surface while viewing the intracellular response by LSCM, ideal for studying cell signaling across the cell envelope and further multiplexing of the data. Fast three-dimensional super-resolution fluorescence imaging^64,65^, using fine-tuned^66^ fluorogenic probes for live cells^67^, shares the same time scale with HS AFM, which would allow rapid single molecule detection, albeit with a higher price tag.

The major advantage of using correlative AFM-QI-LSCM as a routine cellulomic assay is the generation of multiplexed data that reports on vital cell surface properties and virtually any internal biochemical signal from live cells in real-time. In addition to surface architecture, AFM-QI probes surface biochemistry and cellular mechanics, while LSCM offers a window into the cell using fluorescently tagged macromolecules. Tracking the response of three very different cell types to external stress demonstrates the broad applicability of the method, with the possibility of assessing the impact of exposure to virtually any type of stress (e.g. oxidative, ΔΨ, ΔpH) brought on by any number of contaminants (e.g. pesticides, personal care products, toxins), alone or in combination.

## Methods

### Strains and growth conditions

The *E*. *coli* parent strain, WM1074 and its mutant WM2026 with FtsZ-GFP, were kind gifts from Dr. William Margolin^68^. WM2026 and WM1074 were grown on lysogeny broth (LB) at 30 °C. WM2026 was induced with 40 µg/mL IPTG two hours prior to harvest. *C*. *albicans* RSY150 co-expressing tubulin2-GFP (Tub2-GFP) and histone protein B-RFP (Htb-RFP), a kind gift from Dr. Richard Bennet, was grown on yeast-extract peptone dextrose (YPD) medium at 30 °C^69^.

HEK 293 cells, a kind gift from Dr. Mohan Babu, were cultured in Dulbecco modified Eagle’s media (DMEM; Life Technologies) supplemented with 10% fetal bovine serum (FBS: Sigma-Aldrich), glutamine (2 mM) and penicillin (50 IU/ml)/streptomycin (50 µg/ml; Life Technologies). Cells were cultured on petri dishes for several passages. These cells were transferred to a petri dish with Cell-Tak coated coverslips and allowed to attach overnight. Cells were transfected with Cell Light® Tubulin-GFP reagent (BacMam 2.0, Life Technologies), at a concentration of 1 particle/cell according to the manufacturer’s instructions, and imaged after 48 h.

### Minimum Inhibitory Concentration (MIC)

The MIC was determined as described previously for *E*. *coli*^9^. Briefly, an overnight stock culture was inoculated into a series of test tubes with 2,4-D over a wide concentration range (0.1–20 mM) and incubated for 24–48 h. The highest concentration of 2,4-D completely inhibiting growth, taken to be the MIC for *E*. *coli*^9^ and *C*. *albicans*, was used to determine an appropriate 2,4-D concentration for experiments at sublethal levels, with sufficient growth for correlative imaging. For HEK 293, a suitable 2,4-D concentration was selected based on Bukowska *et al*.^70^.

### Sample preparation

Coverslips were cleaned with plasma (Harrick Plasma Cleaner, PDC-32G) or piranha solution as previously described^9^ and stored for up to 2 weeks at room temperature. A 30 µL aliquot of freshly prepared Cell-Tak solution (145 µL of pH 8 NaHCO_3_ buffer, 5 µL of 1 mM NaOH and 5 µL of Cell-Tak) was spread (1 sq. cm) onto the precleaned coverslip, incubated and rinsed. Prior to incubation of samples for imaging, coverslips were made sterile by rinsing with ethanol.

An overnight culture of *E*. *coli* (O.D_600_ ~ 1) was inoculated into 5 mL of fresh LB media and allowed to grow for 2–3 h (O.D_600_ ~ 0.4) to obtain culture at the mid-log phase. Approximately 500 µL of culture was added to the coverslip and incubated (32 °C, minimum of 30 min) in the dark. The samples were rinsed and imaged under PBS (0.01 M, pH 7), LB diluted 1:1 with PBS or in LB media alone. In all cases, the sample was rinsed thoroughly with imaging media and mounted with 500 µL of the same solution onto the AFM stage. The *E*. *coli* WM2026 imaging media contained 40 µg/mL IPTG. Media and PBS were sterile filtered (0.2 µm) and incubated at 30 °C for experiments. Coverslips were glued to the bottom of a custom made petridish using epoxy and placed in the JPK petridish incubator.

An overnight culture of *C*. *albicans* RSY150 was diluted to an OD_600_ of 0.2 and incubated 3–4 h in fresh media at 30 °C with shaking until it reached exponential phase (OD_600_ = 1). These cells were diluted (1:3) with media and incubated on the sterile Cell Tak coated cover slips at 30 °C for 30 min, allowing the cells to attach. Prior to imaging, YPD media was replaced by a solution of 1:1 YPD and PBS.

Transfected HEK 293 cells were grown onto Cell-Tak coated coverslips that had been glued to the bottom of custom made petri dishes and incubated for 48 h at 37 °C in the presence of 5% CO_2_. A nuclear dye (SYTO™ 82, 5 mM in DMSO; Life Technologies) was added to cells to a final concentration of 5 nM, incubated for 15 min and imaged immediately.

For all samples, Cell ROX Deep Red dye (Life Technologies) was added at a concentration of 5 µM, 30 min prior to the addition of 2,4-D. During experiments the sample was maintained at a temperature of 30 °C for *E*. *coli*, and 37 °C for *C*. *albicans* and HEK 293 cells.

### Live-AFM-LSCM

Essential elements of the AFM-LSCM setup are shown in Fig. 1. The entire system is housed on a vibration isolation platform and in a home-built acoustic enclosure. Our AFM-LSCM setup consists of the Nano Wizard 3 AFM (JPK Germany) mounted on an inverted LSCM 780 (Carl-Zeiss) as described previously^34^. The custom designed AFM stage (JPK) was positioned in place of the LSCM stage and the sample mounted as described above. An additional camera, attached to the LSCM front port and connected to the AFM-ECU, produced a DIC image of the AFM tip, laser alignment and sample viewed from below. Initial viewing with the 20× objective allowed laser alignment and assessment of the distance between AFM tip and sample. A drop of the sterile imaging media was slowly placed onto the cantilever and allowed to slide onto the tip to avoid its sudden jump into contact with liquid, which can cause tip damage due to a rapid change in surface tension. The tip was lowered into the liquid, viewed with the 20× objective, positioned on a suitable point of the sample and the AFM laser realigned with the photodetector to optimize signal from the photodiode under fluid.

Subsequent viewing with a 63× oil immersion objective allowed the choice of a single cell which appeared immobile on the front port camera and confocal, but was engaged in cell division (*eg*. FtsZ-GFP ring at the mid cell for *E*. *coli*). Optical calibration (JPK software) precisely positioned the AFM tip in relation to the cell to generate optical overlay of the AFM-LSCM images. The spring constant of AFM cantilevers (Nanoscience, model no: HYDRA4V-100N), having a low nominal spring constant (k = 0.08 N/m), were calibrated (k = 0.05 ± 0.03 N/m in imaging media) using the thermal noise method^71^ prior to each data collection.

One challenge in live microbial imaging was to locate a suitable, actively dividing, but immobile cell for imaging by AFM. A force map at low resolution (20 × 20 pixel, 15 µm square) served as a guide for determining the location of cells with respect to tip position. Areas with high pixel intensity in the force map height image were centered and imaged at high resolution, and a very small field of view imaged by QI to obtain a complete map of Young’s moduli and adhesion values. Z-length, set point and imaging speed were all adjusted on the fly during QI imaging to minimise noise.

Thorough sample rinses ensured that the majority of planktonic bacterial cells were removed, but with an average *E*. *coli* doubling time of 20 min the imaging environment altered rapidly. The same cell could be repeatedly imaged, simultaneously with AFM and LSCM for 2–3 h, after which time image quality deteriorated from quickly dividing planktonic cells which obstructed cantilever movement and laser alignment. At this point, the AFM head was removed, the sample washed gently several times with fresh media, and imaging resumed as previously described. After replacement of the AFM head onto the LSCM stage, the optical overlay ensured that a cell of the same progeny, which after generations of division (several hours) often appeared as a mini colony (Fig. S2), could be selected for additional imaging. Since the collection of a single AFM image can take up to 20 min, AFM images of cells showed dramatic changes from one image to the next. The addition of fresh media every few hours ensured that cells had sufficient nutrients and that toxic waste was removed in adequate time. A similar approach for choosing a suitable cell was successful for both *C*. *albicans* and HEK 293 cells. Samples of the eukaryotic *C*. *albicans* and HEK 293 cells did not suffer from fast doubling times, overcrowding and planktonic cells as for the prokaryote *E*. *coli*.

A Z-length of 3 µm, set point 0.5 nN and an extend/retract speed of 100 µm/s produced high resolution images with the least noise in most cases for bacteria. The Z-length and set points were increased to 7 µm and 0.6 nN for yeast to accommodate their large height and stiff surface. Based on their relative fragility, HEK 293 cells were imaged with a Z-length of 5 µm and a lower set point (0.2–0.3 nN). Imaging time is affected by the Z-length, and the extend/retract speed. Confocal time lapse images were collected directly before, after and in some cases during QI imaging. Confocal imaging could be performed during QI imaging, provided all the confocal parameters were set prior to the start of imaging, as any mechanical movement of the confocal during imaging could cause the AFM tip to crash or produce large noise in the QI image.

Multicolor confocal images were obtained using the lambda mode and by selecting a suitable range of excitation wavelengths. The channels were later separated during image processing using the linear unmixing function (Zen Blue). ROS intensity was measured using the intensity measurement function for the red channel after subtracting the background intensity (Zen Blue).

The *E*. *coli* WM1074 was imaged by AFM-QI alone, while *E*. *coli* WM2026, *C*. *albicans* RSY150, and HEK 293-tubulin-GFP were imaged by AFM QI-LSCM (λ_ex/em_ GFP; 488/510, RFP; 554/591; Cell –ROX 640/665, Syto 82; 541/560).

### AFM-LSCM data processing

AFM and LSCM images collected simultaneously or at similar times were overlaid using Photoshop 11. LSCM images were processed for contrast and digitally enlarged to coincide with the AFM height image. QI force curves from each pixel of a force map (128 × 128 image) were processed (JPK software) using baseline adjust (adjust for deflection offset in the y scale), contact point adjust (offset adjust of x-scale) and a height correction to determine the vertical position of the tip derived from cantilever bending. Adhesion was calculated by subtracting the lowest point of the retract curve from the baseline and the apparent Young’s moduli, reflecting cell envelope elasticity, was estimated from a Hertzian fit of the approach curve (JPK software). QI data, only at the centre of the cells, was used for final quantification to eliminate edge artifacts that arise near the periphery. Surface roughness, measured from QI height images at the mid-point of the cell, has been described previously^9^. Force curves from QI images were batch processed according to Bhat *et al*.^34^. For each cell type, a minimum of ten cells from three biological replicates were imaged and analyzed. Each image (128 × 128 pixels) produced 16,384 force curves, however a small area (~200 × 200 nm for bacteria, ~500 × 500 nm for *C*. *albicans* and ~2 × 2 µm for HEK 293) at the centre of the cell was chosen to process force curves, excluding tip artifacts near the edges.

Histogram data was exported from the JPK software for statistical analysis and plotting. Data were statistically evaluated by unpaired student’s t-test and one-way ANOVA (Graph Pad Prism 5.01).

## Electronic supplementary material

Supplementary data

Movie S1
