# Supplementary material for: Correlative atomic force microscopy quantitative imaging-laser scanning confocal microscopy quantifies the impact of stressors on live cells in real-time
Source: Sci Rep. 2018 May 29;8:8305. doi: 10.1038/s41598-018-26433-1 (PMC5973941; doi:10.1038/s41598-018-26433-1)
Supplement: Supplementary file 1 — Supplementary data [file 41598_2018_26433_MOESM1_ESM.pdf]

Correlative atomic force microscopy quantitative imaging-laser scanning confocal microscopy quantifies the impact of stressors on live cells in real-time

Supriya V. Bhat[a], Taranum Sultana[a], André Körnig[b], Seamus McGrath[a], Zinnat Shahina[a], Tanya E. S. Dahms[a]*

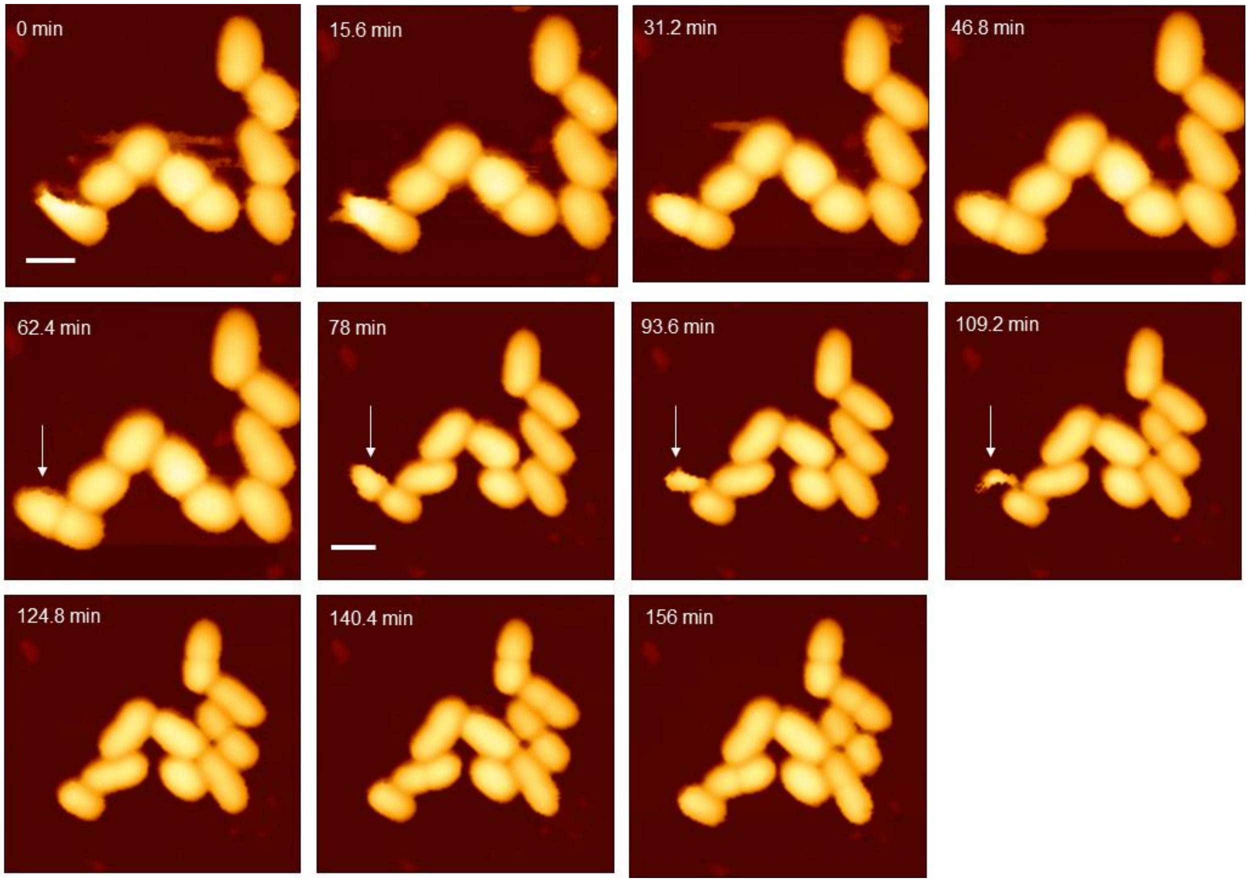

Figure S1. AFM-QI time lapse images showing various stages of *E. coli* cell division. AFM-QI images, taken every 15.6 min in 0.05 M PBS, show cell elongation, constriction at the mid-cell, and separation of daughter cells. White arrows show a cell slowly detaching from the surface immediately after division and eventually becoming fully planktonic. Scale bar, 1 μm.

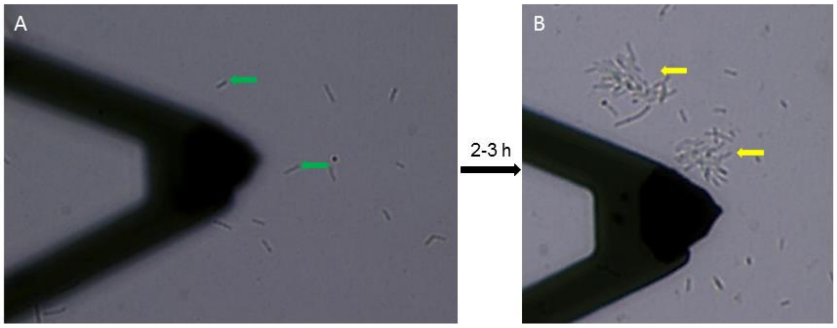

Figure S2. DIC image of the AFM-QI-CLSM field of view showing the V-shaped AFM cantilever and bacterial cells. The cantilever is out of focus and the bacteria in focus for image A and the opposite for image B. Individual cells (green arrows) on fresh samples are shown in A and micro colonies (yellow arrows) originating from those cells following several hours of growth are shown in B.

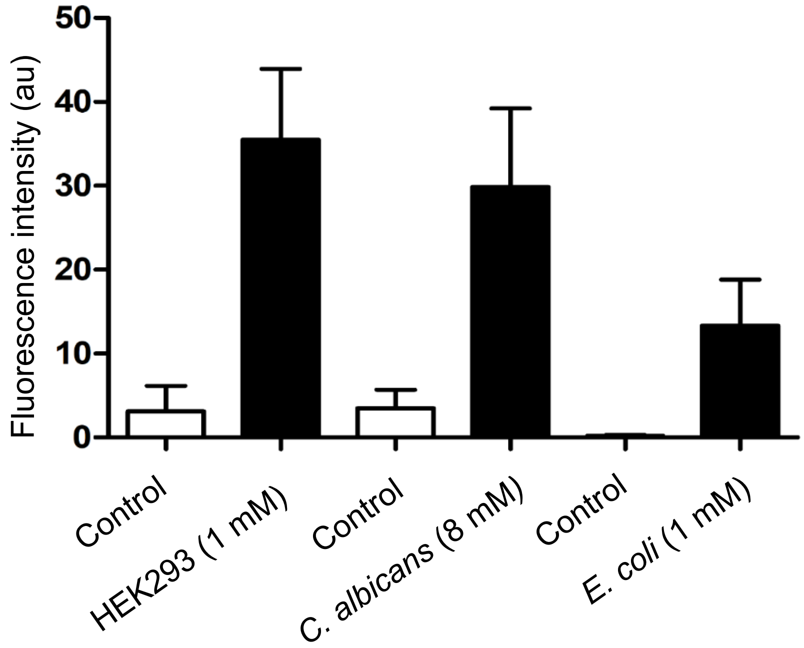

Figure S3. Plot of Cell ROX Deep Red ($\lambda_{ex/em}$ 644/665) fluorescence intensity versus cell type. A significant ($p < 0.0001$) increase in ROS was observed for HEK293, *C. albicans* and *E. coli* during 2,4-D exposure compared to their respective controls.

W2026_3D_1fps_vid.avi

Movie S1. Movie created from AFM-QI time lapse images taken every 15.6 min in 0.05 M PBS, showing various stages of *E. coli* cell division, including cell elongation, constriction at the mid-cell, and separation of daughter cells. Following division, one daughter cell detaches from the sample surface, becoming planktonic.
